# Supplementary material for: Photoluminescent Polymer Aerogels with R, G and B Emission
Source: Int J Mol Sci. 2022 Dec 15;23(24):16004. doi: 10.3390/ijms232416004 (PMC9785137; doi:10.3390/ijms232416004)
Supplement: Supplementary file 1 [file ijms-23-16004-s001.zip › ijms-2059336-supplementary.pdf]

Supplemental Information

1. Thermal Investigation

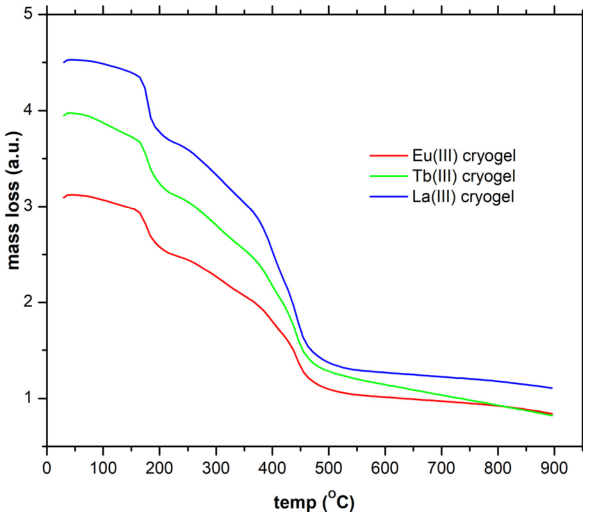

Figure S1. Thermal analysis of the prepared Eu(III), Tb(III) and La(III) aerogels.

2. XPS Investigation

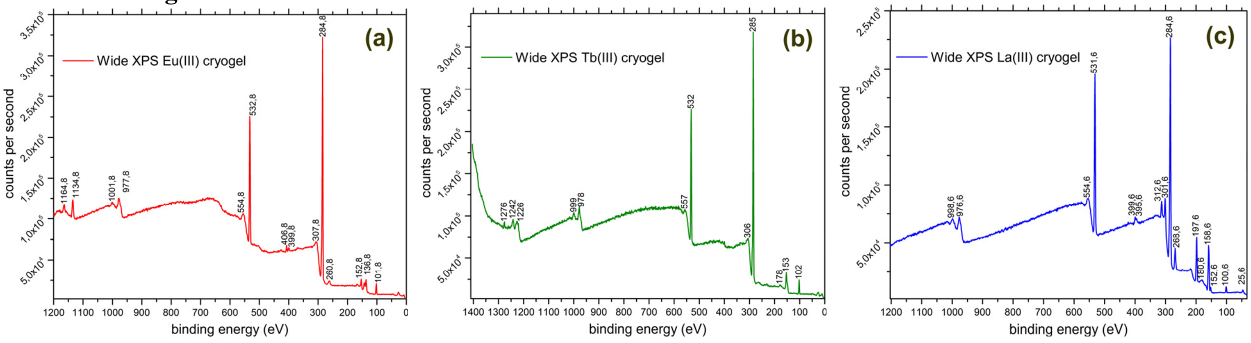

Figure S2. Recorded wide XPS spectra for (**a**) Eu(III) aerogel, (**b**) Tb(III) aerogel, (**c**) La(III) aerogel.

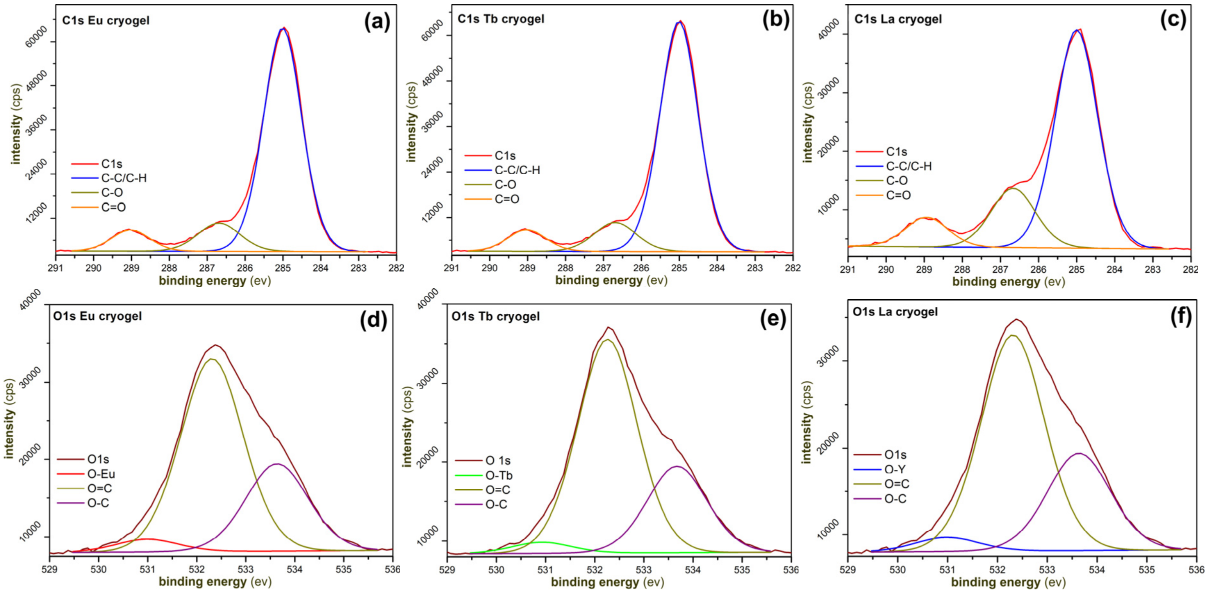

Figure S3. Recorded high resolution XPS spectra C1s (**a**) Eu(III) aerogel, (**b**) Tb(III) aerogel, (**c**) La(III) aerogel and O1s (**d**) Eu(III) aerogel, (**e**) Tb(III) aerogel, (**f**) La(III) aerogel.

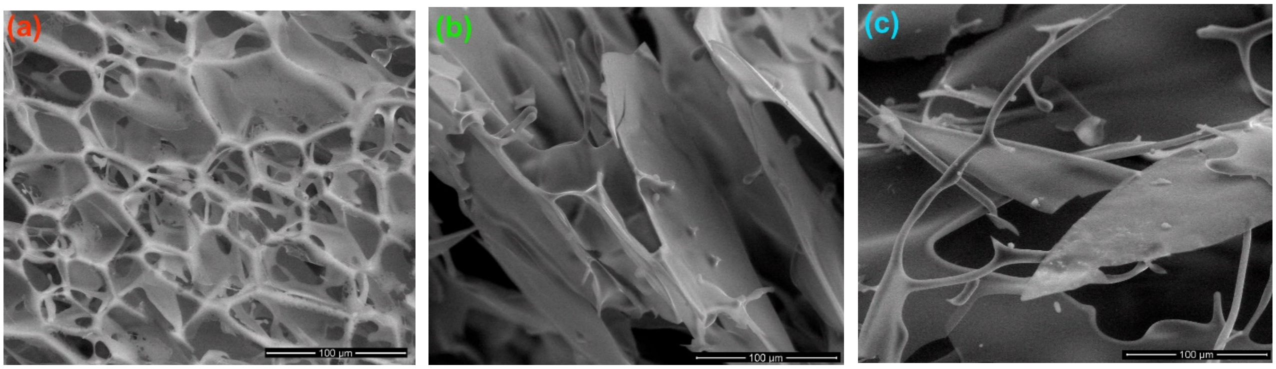

Figure S4. SEM micrograph of the prepared (**a**) Eu(III) aerogel, (**b**) Tb(III) aerogel, (**c**) La(III) aerogel at higher magnification (1000x).
